# The Inside Out? Views of Young People, Parents, and Professionals Regarding Successful Secure Residential Care

**DOI:** 10.1007/s10560-016-0473-1

**Published:** 2016-11-15

**Authors:** Annemiek T. Harder, Erik J. Knorth, Margrite E. Kalverboer

**Affiliations:** Department of Special Needs Education and Youth Care, Faculty of Behavioral and Social Sciences, University of Groningen, Grote Kruisstraat 2/1, 9712 TS Groningen, The Netherlands

**Keywords:** Adolescents, Behavior change, Treatment skills, Secure residential youth care, Success factors of treatment

## Abstract

Although adolescents often seem to improve in their functioning during residential care, there still is little knowledge on what factors are important in achieving these changes. The present study aims to identify the care factors that are important for adolescents’ behavior change during secure residential care. We conducted in-depth interviews with eight adolescents, their parents, eight group care workers and seven teachers concerning their in-care experiences. Both adolescents and parents commonly attributed changes during secure residential care to the treatment environment. Group care workers and teachers did not have a clear, consistent view on the treatment aspects causing positive change with the adolescents. According to the adolescents, good professionals apply a fine balance between rules and freedom, show empathy and are available for support. The view of parents corresponds to this image. Although group care workers are perceived as available for support, adolescents tend to make little use of this help if they experience personal problems during care. The results highlight the importance of responsiveness of secure residential care professionals to the needs and perspectives of adolescents and parents.

## Introduction

Compared to their peers utilising other types of out-of-home care (i.e. foster care or family-style group care), adolescents in residential youth care exhibit more serious behavior problems (Leloux-Opmeer, Kuiper, Swaab, & Scholte, 2016). These problems are often reflected in externalising and disruptive behavior. In many cases, their parents are no longer able to handle them, thus rendering residential services appropriate and necessary (Harder, 2011). Young people in residential youth care receive 24-h care, and they are supervised for at least several days each week. In the Netherlands, about 11–14% of the nearly 205,000 children and adolescents in child and youth care uses residential care services (Knorth, 2005), which is a small group within the total group of 4.9 million young people between the ages of 0 and 25 in the total population of 16.3 million people (CBS, 2009).

Secure residential care is the most intensive or restrictive form of residential youth care. In this type of care, young people between the ages of 12 and 23 years, who are regularly placed under coercion, reside in a secure environment. In the Netherlands, secure residential care is provided within juvenile detention facilities and Youth Care Plus institutions. Although these institutions operate within different legal frameworks (criminal and civil law, respectively), both can be regarded as secure care facilities (Harder, 2011). In practice, both types of institutions in the Netherlands focus primarily on the care and treatment of adolescents with similar antisocial and disruptive behavior (Goderie, 2004). Besides a focus on treatment of the admitted adolescents, secure residential care is also aimed at protecting the community against the undesirable behavior of these young people. Therefore, Lemmond and Verhaagen (2002) describe secure residential youth care as a type of care that “..allows for intensive focus on treatment, strict control of the youth’s environment, and protection for the community…” (p. 2).

Whereas adolescents often improve in their functioning during secure residential care (Knorth, Harder, Zandberg, & Kendrick, 2008) they still regularly show problem behavior after their departure, for example in terms of delinquent behavior or recidivism (Abrams, 2006; Lipsey, 2009; Orlando, Chan, & Morral, 2003). For one thing this can be explained by the severity and complexity of the problems that are often encountered in these adolescents (Bullock, Little, & Millham, 1998), for another by limitations in the available services, such as a treatment approach that is coercive instead of therapeutic (cf. Lipsey, 2009).

### ‘What Works’ in Residential Youth Care

Regarding successful treatment, research has yielded several guidelines that are often referred to as *‘*what works’ principles: the ingredients that proved effective in treatment (Carr, 2009). A line of research which is relevant here concerns studies focusing on what works in (secure residential) youth care. These studies often refer to the so called non-specific and specific treatment factors. Non-specific or common treatment factors are those factors that affect the services offered, regardless of the target group or the type of services. Specific treatment factors are only operating with regard to certain types of intervention and certain target groups (e.g., Duncan, Miller, Hubble, & Wampold, 2010).

Non-specific factors, such as client factors and client-therapist relationship factors, are considered to be the most important predictors of outcomes in child and youth care (Carr, 2009; Karver, Handelsman, Fields, & Bickman, 2006). Client factors consist of the factors that are part of the client, such as the severity of the problems and the clients’ strengths and motivation for treatment. Relationship factors refer to the therapeutic relationship, which is most commonly defined as an emotional and/or a cognitive connection between a client and therapist in terms of agreement on the tasks and goals of treatment (Karver, Handelsman, Fields, & Bickman, 2005). Important therapist factors related to the client-therapist relationship are, for example, a client-centered attitude, communication- and listening skills, and self-reflection (Ackerman & Hilsenroth, 2003).

Specific treatment factors that are considered to be important for successful outcomes in (secure) residential youth care include a supportive, safe environment and specific therapeutic interventions focusing on the individual needs of the adolescents during care (Boendermaker, Van Rooijen, & Berg, 2010; Clough, Bullock, & Ward, 2006; Knorth et al., 2008). Family-focused interventions are also considered important for improving residential care outcomes (Geurts, Boddy, Noom, & Knorth, 2012), although Glough et al. (2006) emphasize that whether and how families can be involved in the care process should be assessed for every individual child, because for some children the involvement of family might mainly have negative consequences.

### Non-specific Treatment Factors in Secure Residential Care

Because adolescents in secure residential care are regularly placed under coercion, often unaware of their problems and resistant to change (i.e., showing a lack of motivation for treatment), non-specific client and relationship factors seem to be especially important for achieving positive outcomes in this care context (cf. Van Binsbergen, Knorth, Klomp, & Meulman, 2001). For example, studies in secure residential care have found that adolescent’s motivation to change is associated with successful outcomes in terms of retention in care (Orlando et al., 2003) and adolescent’s treatment satisfaction (Harder, Knorth, & Kalverboer, 2012). Research indicates that adolescent’s motivation for change can be developed during secure residential care, although a functional therapeutic relationship to promote this motivation is more difficult to establish with adolescents showing serious psychopathology than with adolescents showing less serious psychopathology (Van Binsbergen et al., 2001).

Several other studies also demonstrated the apparent importance of a good adolescent-staff relationship during secure residential care. It is found to be associated with successful outcomes in terms of adolescent’s satisfaction about treatment (Harder et al., 2012), perceived likelihood of success after leaving care (Marsh & Evans, 2009), and recidivism (Florsheim, Shotorbani, Guest-Warnick, Barratt, & Hwang, 2000). Moreover, a good relationship between adolescents and both group care workers and teachers during care is found to be strongly associated with good communication skills of these staff members in contact with adolescents (Harder, Knorth, & Kalverboer, 2013). For example, the ability of teachers to handle poor academic motivation and externalizing behavior problems of adolescents seems to be specifically important for the improvement of adolescent’s academic achievement during secure residential care (Harder et al., 2014).

Although studies do indicate that adolescents in relatively strong relationships with staff members show more improvement on various outcome measures, there still is little exact knowledge on how positive outcomes with adolescents can be achieved during secure residential care. More specifically, it is largely unknown how client-staff relationships can be drawn up to promote improved outcomes. Moreover, there is limited work on clients’ and/or staff’s perceptions of interventions in secure residential care (Schubert, Mulvey, Loughran, & Losoya, 2012). Likewise, to the best of our knowledge, there are no studies on parents’ perceptions of secure residential youth care, despite the fact that the involvement of the adolescent’s family is often considered important for achieving successful outcomes after leaving care (Geurts et al., 2012).

### Aim of this Study

The aim of the present study is to identify the care factors that are important for achieving behavior change with adolescents during secure residential care. We therefore use perspectives from the inside: i.e. adolescents, their parents, group care workers, and teachers within a secure youth care institution. The central question addressed in this article is as follows: Which aspects of the services and education provided within a secure residential youth care institution do adolescents, parents, group care workers, and teachers consider important for a positive behavior change with the adolescents during their stay in the institution? This question can be further elaborated into two sub questions:

- What do adolescents, their parents, group care workers and teachers consider important factors for a positive behavior change of adolescents during secure residential care?
- What do adolescents and parents consider being characteristics of good group care workers and teachers within a secure youth care institution?

## Method

### Participants

In all, six boys and two girls with a mean age of 17 ranging from 15 to 20 were interviewed. The mean length of their stay in the institution at the time of the interview was eleven months, ranging from six weeks up to two year. With one exception, all of the boys were being held at the institution under criminal court order. Both girls were being held at the institution under civil court order. Reasons for admission to the center include criminal offences (e.g. stealing cars, fighting with another person), running away (from home or foster care), substance use (e.g. soft drugs), problems at home and financial problems. Although the young people mentioned these reasons for their admission themselves, six of them did not think that they had a problem at the moment of the interview. One of the boys mentioned that he should work at things, such as school and soft drug use, but that those things are “not serious, those people make it much more serious”.

In general, the ethnicity of the adolescents was Dutch: six of the eight adolescents (75%) had been born in the Netherlands and were of Dutch descent. Two boys were of non-Dutch descent; one of them was born in the Netherlands and the other was born in a different country. Before their admission to the facility, three boys had been living with their parents, while the other five adolescents had already been living in a residential facility, including other residential groups at the secure care institution. All of the adolescents had received other types of care before admission, including substance abuse treatment, stays at various residential facilities, home-based social services and stays with various foster families.

The parents of seven adolescents, i.e. two biological mothers, two biological father and four couples, participated in the interviews. From one girl, both her biological father and her biological mother and stepfather were interviewed. Two couples consisted of a biological mother and a stepfather, one couple were foster mothers and another couple consisted of a biological mother and father. The ages of the parents, excluding the two stepfathers, ranged from 31 to 51, with an average of 41.

The group care workers who were interviewed included four men and four women. The age of the group care workers ranged from 28 to 51, with an average age of almost 36. Most of the group care workers were of Dutch descent (63%). The length of time that they had been employed at the secure care institution ranged from almost one year to just over 6 years. Half of them had been working in the institution for 3 or 4 years.

Most of the seven teachers who were interviewed were male (71%), with ages ranging from 29 to 55. The average age of the teachers was 42.5. The majority of the teachers were of Dutch descent (71%). The length of time that the teachers had been employed within the secure care institution ranged from 1.5 to 8 years. Six of the seven teachers had been working in the institution for four years or longer. Compared to the group care workers, the teachers who were interviewed had been employed at the institution for longer.

### Research Design

The present study is part of an exploratory study that was conducted during a research project within a secure residential youth care institution in the Netherlands (Harder, 2011). In addition to case-file research and participant observation in residential groups and classes, this study included in-depth interviews with adolescents, their parents, group care workers, and teachers. The present study is based on data obtained during the interviews that were all conducted by the first author of this study.

### Procedure

Based on the organization’s client administration system and data regarding types of accommodation in use, eight young people were selected at random for in-depth interviews: four adolescents who had been sentenced to the detention facility and four who had been placed under civil order. They were selected using a digital program, which provides a display of the placement data for all adolescents in the facility. Only those who had a mastery of the Dutch language were approached for interviews.

Before the adolescents were approached, the treatment coordinator for the residential group in question was contacted. After that, the researcher had telephone contact with the group care workers at the residential groups. Both these contacts were made very easily, because the researcher was familiar with professionals in both facilities by previously conducted participant observations in different residential groups. After the phone calls, the adolescents were individually approached by the researcher during a short conversation at the residential group, in which the researcher provided the adolescent with information about the aim and contents of the interview, and asked the adolescent whether he or she wanted to participate. Because one of the adolescents approached did not wish to participate, another was selected at random. Six adolescents were interviewed in separate interview rooms, and two were interviewed at the residential group without the presence of others.

The parents (or foster parents) of all participating adolescents were also approached for interviews. The first request was sent in the form of a letter. About a week thereafter, the parents were contacted by telephone in order to invite them to participate. The parents of one boy could not be reached. All of the other parents who were approached participated in the interview. The interviews with parents were held either in their homes or at the secure care institution. Since one of the mothers had difficulties with understanding the Dutch language, some of the information from that interview was missing.

Group care workers were selected from the eight residential groups in which the researcher had initially conducted participant observations. One group care worker was selected from each residential group. In selecting care workers, there was a preference for care workers who had worked at the facility for a longer time, as they had gained the most experience and were thus more likely to have formed the best image of the institution.

Nearly all of the teachers from the classes in which observations had been conducted were approached for interviews. The teachers were selected with a preference for those who had been working at the facility for longer periods. From the detention facility, five teachers from the five educational sectors (i.e., one theoretical sector of preparatory secondary vocational education and four practical sectors: metal, construction, health and welfare, and consumer technology) were selected and interviewed. In the Youth Care Plus facility, teachers were selected from classes including adolescents who had been placed under a civil order. Due to the holiday period and delayed observation in the classes, only two teachers from this school were interviewed. In all, therefore, seven teachers participated.

Audio recordings were made (with permission) during almost all of the interviews with adolescents and parents. With group care workers and teachers notes were taken, because audio recorders were not available during those interviews. For verification, a written report of the interview was given to each participant. We only received feedback from the couple of foster mothers to make small corrections in the report. In the reports for the adolescents a fictitious name was used for each of them, which was chosen by him or her directly after the interview. The study conforms to internationally accepted ethical guidelines. All respondents gave verbal consent to participate in the study.

### Setting

The setting of the study is a secure residential youth care institution serving adolescents between the ages of 12 and 23 years. Adolescents are almost always placed in the institution involuntarily. The primary reason for placement is intolerable behavior, including delinquency. The institution consists of a juvenile detention facility for criminal juvenile delinquents, as well as a secure residential care center (‘Youth Care Plus facility’) for adolescents with serious emotional and behavioral problems placed under a civil order. The detention facility functions in the context of juvenile criminal law and the secure residential care center within the context of civil law. Although these facilities have different legal contexts, both these facilities can be considered as secure residential youth care, because in practice both are locked facilities which offer care and treatment for adolescents who often show similar antisocial and acting-out behavior problems. Moreover, the aim of both facilities is to offer care and treatment to the adolescents to improve their functioning. A secure environment is maintained within both facilities, such that nearly all of the adolescents are under supervision 24 h/day, seven days per week. In many cases, they are not allowed outside of the facility without supervision. Key components of the services provided include the residential community and school attendance, often in the internal school.

### Interviews

The structured interviews were prepared within the framework of the research project, with the goal of recording the experiences and views of adolescents, parents, group care workers, and teachers with regard to residence or employment in the institution. The contents of the interview were partly based on a literature review of empirical studies on the quality and outcomes of residential youth care (Harder, Knorth, & Zandberg, 2006). The interviews all had a similar structure and generally the same contents, which made it possible to compare the different perspectives.

For the present study, we used respondents’ answers to both close-ended (e.g. ‘Does you stay in the institution lead to a change on you?’) and open-ended questions (e.g. ‘If so, what caused that change?’) regarding two specific topics. The first topic was included in the interviews of all respondents and concerns the behavior change of adolescents during their stay in the facility (e.g. ‘Which treatment aspect has most effect on the young people?’). The second topic covers characteristics of good group care workers (i.e. ‘What is a good group care worker according to you? What should he or she do?’). This topic was only included in the interviews of the adolescents and their parents. In addition, we included adolescents’ perspectives about characteristics of good teachers (i.e. ‘What is a good teacher according to you? What should he or she do?’) and the availability of care workers for support of the adolescents (i.e. ‘Do group care workers help you with your problems? If they do, for which problems do they do so? And how do they help you?’).

### Data-Analysis

The data were analyzed using inductive strategies (Patton, 2002). First, the written interview reports for all respondents were systematically organized by the first author according to the main topics and questions in the structured interviews. As a result, the interview reports for each respondent group (i.e. adolescents, parents, group care workers and teachers) included exactly the same headings referring to the same main topics in each interview. Then the first author selected the text parts with regard to the relevant topics behaviour change and characteristics of good professionals out of the written reports for each respondent group separately. To decide what information belonged to behaviour change and to characteristics of good professionals the interview questions were used as a guiding principle. In accordance with the specific interview questions concerning each topic, the first author placed the corresponding text parts from the interview report of each respondent one below the other by using Microsoft Office Word. Then the first author read the text parts for each topic several times to identify the themes mentioned by each respondent group. Finally, key concepts that directly emerged from the interview reports were identified by carefully examining and highlighting key words in the interview text parts.

## Results

### Behavior Change During Care

All six adolescents who mentioned that their stay in the institution changed them also mentioned that they thought that they should change. With regard to this change, one of the girls mentioned “I will never change in the way that they would like”, referring to the difference between her own perspective on treatment goals and that of the care professionals in the secure residential care center. The change mentioned by the six adolescents was attributed to their environment: their stay in the institution, the people surrounding them during care, a stay in the police station, and the care received. The other two adolescents, both boys, neither mentioned that they changed during care nor thought that they should change.

The parents of four adolescents perceived changes with their child during care. They were divided in whether they thought change was necessary. The changes perceived by these parents were both attributed to the environment (i.e., the group care workers and day-to-day interaction; the rules, regularity, structure and the absence of the wrong friends during secure residential care), and to the adolescents themselves (i.e., the adolescents’ understanding of their own situation and their age).

Group care workers had different ideas about the aspects of treatment that could cause positive change with the adolescents. They mentioned talking/conversations with the adolescents, the overall treatment approach (i.e., by care workers’ actions, care structure, and clarity for the adolescents to know where they stand), acquiring confidence with the adolescents, the group care workers and other care professionals, adolescent’s attendance at school, and the mutual contact between adolescents.

Teachers also mentioned different treatment aspects that could cause positive change with the adolescents, including the overall treatment approach (i.e., compulsory attendance at the residential group and school), deprivation of freedom, learning practical skills (e.g., hygiene), independence, and the implementation of rules/discipline, adolescent’s experiences of success and the use of positive rewarding, consequent behavior of staff members, good individual coaching of adolescents by group care workers, and specific types of individual treatment. One of the teachers indicated that he did not have a clear view on the treatment process and therefore could not mention which aspects could cause change with the adolescents.

Five group care workers also mentioned which aspect they considered as having the most effect on the young people. Both peace and regularity during care and the adolescents’ motivation for change and understanding of their own individual problems were mentioned twice, followed by the contact with other adolescents. Teachers also named the mutual contact between adolescents as having the most effect on the young people, as well as the staff members that keep in closest contact with the adolescents, and the cooperation between different staff disciplines.

### Characteristics of Good Professionals

#### Group Care Workers

The eight adolescents mentioned several characteristics of a competent group care worker. In addition to being someone with whom they can *“*just talk”, who is *“*just normal*”,* does what he or she must do, and who can interact with adolescents in a “normal” fashion, adolescents named several skills (see Table 1).

**Table 1:** Skills of a good group care worker according to adolescents (*n* = 8)

Main skill	Description by the adolescents
1. Authority: balance between rules and freedom (*n* = 5)	Does not take any more liberties away from adolescents than they have already lost and follows the rules without being extremely strict (e.g., with previous warning)
	Allows a certain amount of space for them to solve their own problems amongst themselves
	Is not a *“youngster”* telling you which way to turn
	Does not actually apply the rules
	Does not do act according to the rules all the time, should not be too authoritarian and has independent judgment
2. Empathic (*n* = 5)	Empathizes with the situations of others
	Someone who also has been through a lot him- or herself
	Is genuinely involved with young people
	Is not too quick to judge
	Is sociable and gets along with everyone
3. Available for help/support (*n* = 4)	Arranges things for young people and helps young people to make progress
	Should be there for you and demonstrates that he or she is available
	Finds a balance between working in the office and being present within the group
	Divides his or her attention well and notices everyone within the residential group
4. Careful (*n* = 2)	Should pay attention to the (household) chores
	Is not forgetful or chaotic
5. Listening well (*n* = 2)	Is listening well
	Truly understands what adolescents mean
6. Reliable (*n* = 2)	Does not put everything in writing
	Is thrustworthy
7. Honest (*n* = 1)	Is being honest
8. Resistant to stress (*n* = 1)	Is resistant to stress

It was more difficult for the parents to characterize a good group care worker than it was for the adolescents. Nevertheless, the parents of five adolescents did list several characteristics. Just as their children, parents most often mentioned that group care workers should not engage in any display of power, but should possess a natural, innate authority. For example, good group care workers are straightforward and firm with adolescents, and they are capable of indicating their own boundaries. Second, they mentioned empathy: a group care worker should be able to anticipate the feelings of adolescents, empathize with them and to have an understanding of their problems. Moreover, a good group care worker should be someone who can accept adolescents as they are and who can treat all children equally and feel affection for these young people. The parents also identified sincere commitment and a sense of humor as characteristics of good group care workers.

#### Teachers

As with group care workers, according to the adolescents good teachers have a number of characteristics (see Table 2).

**Table 2:** Skills of a good teacher according to adolescents (*n* = 8)

Main skill	Description by the adolescents
1. Authority: balance between rules and freedom (*n* = 5)	Does not make students work the whole day, but allows them to take breaks. Need not monitor everything constantly, although should ensure that the classroom remains quiet and tidy, and that everything runs smoothly
	Is neither too strict nor not too tolerant in implementing rules
	Should not *“complain too much that you ought to get to work”*
	Is not a strict person *“who can’t stand anything”*
	Is nice and not too strict
2. Empathic (*n* = 4)	Takes the initiative towards having a conversation and has the ability to engage in real discussion with students
	Develops good relationships with his or her students
	Interacts with the class in order to keep the students interested
	Adjusts to the level and/or personal characteristics of individual students
3. Supportive (*n* = 2)	Is willing to explain things more often if you do not understand
	Provides opportunities to students
4. Expertise (*n* = 1)	Knows what he or she is doing
5. Humor (*n* = 1)	Is someone with whom you can laugh
6. Inspiring (*n* = 1)	Is interesting to listen to
7. Patient (*n* = 1)	Is patient with you

Just as for group care workers, the adolescents most often mention that teachers should apply a fine balance between rules and freedom in the classroom, followed by showing empathy and being supportive.

#### Support for Personal Problems

In response to the question of whether group care workers helped the adolescents with their problems, all adolescents noted that group care workers could help them when they experienced problems. For example, one of the girls mentioned: “Some of the group care workers here really do try to help you with things.” However, only one boy said that care workers actually helped him with his problems. Some group care workers (particularly the nice ones) did help him with his problems (e.g., his habit of smoking joints). They kept on top of things in this regard, which he didn’t like. They were always available to him, and they wanted to know whenever anything was bothering him.

Three boys did not have a need for help from group care workers. Another boy indicated that adolescents are able to talk with the care workers if they are having problems, but that they rarely helped him when there were problems. The sixth boy that was interviewed mentioned that he could express his problems to group care workers, but then nothing was done with it. One girl indicated that she could “ask for a conversation” with a group care worker if she was having problems or when something was bothering her. However, they did not take the initiative to approach her. She commented: “They usually don’t notice if something’s bothering you. If you say, ‘It’s nothing,’ then they assume everything’s pretty much okay.” The other girl mentioned that not all group care workers were available if there were problems and that she could not rely on their support at all times.

## Discussion

The present study aimed to identify the care factors that are important for achieving behavior change with adolescents during secure residential care by focusing on the in-care experiences of adolescents and their parents, group care workers and teachers. A first key finding is that both adolescents and parents commonly attributed behavior changes of adolescents during secure residential care to the adolescent’s environment. This might suggests that although most adolescents thought that they themselves should change, they adapted their behavior during care to satisfy external demand (Ryan & Deci, 2000) since they knew what was expected of them and how they should behave (cf. Abrams, 2006). Such an external regulatory process can explain why it is difficult to sustain positive behavior changes áfter secure residential care, since behaviors that are based on an own decision (autonomy) and performed voluntarily (i.e., in the absence of material rewards or external constraints) are necessary to achieve an actual behavior change (cf. Ryan & Deci, 2000).

Given that adolescents themselves consider their own motivation for behavior change as a key element of the change process in secure residential treatment (Henriksen, Degner, & Oscarsson, 2008), the exact role of their own responsibility should receive additional attention in both research and practice (cf. Englebrecht, Peterson, Scherer, & Naccarato, 2008). Using the self-determination theory as a theoretical frame of reference is recommended here, because this theory has received much empirical support in other research fields and is highly relevant, but strikingly neglected in secure residential treatment (cf. Vansteenkiste & Sheldon, 2006). This theory can offer guidelines in further studying the role of the environment versus the own responsibility in achieving positive behavior change with young people during (and after) care.

A second key finding is that both group care workers and teachers did not agree on which treatment aspects could cause positive change with the adolescents. Aspects that were considered as having the most effect on the adolescents were, for example, the peace and regularity during care, adolescents’ motivation for change and understanding of their own individual problems, and the contact with other adolescents. However, care workers and teachers did not appear to have a correspondingly clear and consistent view on how changes could or should be achieved with the adolescents. This corresponds to research showing that, in general, group care workers often act according to their own ideas and personal styles (Andersson & Johansson, 2008; Knorth, Harder, Huyghen, Kalverboer, & Zandberg, 2010). However, agreement on the applied treatment approach among care professionals is found to be associated with successful residential care outcomes (Department of Health, 1998). In achieving such agreement, the management and particularly the heads of secure care facilities might play an important role by disseminating a clear view on how to achieve positive behavior changes with the adolescents (Department of Health, 1998; Hicks, Gibbs, Weatherly, & Byford, 2008). Therefore, the potential impact of supervision of care professionals by managers and directors in secure residential care facilities needs further clarification in future research studies.

A third key finding is that all of the adolescents interviewed in this study were able to provide a clear description of good group care workers: they should apply a fine balance between rules and freedom at the residential group, be available for support, possess good listening skills, and be reliable and empathic. The view of parents corresponds to this image. According to the adolescents, good teachers should also apply a balance between rules and freedom, followed by possessing good communication skills and be supportive. The few other studies on adolescent’s views regarding characteristics of good professionals in secure residential care reveal similar results. For example, the adolescents addressed in a study by Van der Vlugt and De Jong (2005) consider good listening skills important, as well as involvement and the ability to “really talk” with them. A more recently published study has shown that adolescents in secure residential care consider the following characteristics of both group care workers and teachers as important: demonstrations of involvement and commitment, a respectful attitude, and the ability to provide clarity, to relate to adolescents, to ensure good contact, to provide feedback, and to stand up for them (Harder et al., 2013).

These findings provide a clear overview of the skills that professionals in secure residential care should possess. They also offer practical suggestions for skills training for both group care workers and teachers. Since several studies found evidence for the association of good professional’s interaction skills with successful outcomes in secure residential care (Harder et al., 2012; Van Dam et al., 2011), we recommend future research to focus on expanding our knowledge on how client-staff relationships can be drawn up to promote improved outcomes of secure residential care. For instance, the research field can contribute to this knowledge by focusing on the effectiveness of training programs for professionals, which is largely a neglected area of research in (secure) residential youth care (cf. Crosland et al., 2008; Harder et al., 2013).

A fourth key finding of the present study is that adolescents noted that group care workers were able to help them when they had problems, but also that they tended to make little use of this help. This result corresponds to other research on the perceptions of adolescents in Dutch juvenile detention institutions, in which adolescents stated that service agents should be more alert to signals and requests for help (which are often obscured) (Van der Vlugt & De Jong, 2005). In correctional facilities in the United States, there also often seems to be a discord between juvenile male offenders’ perspectives and the applied treatment approach by staff (Abrams, 2006). With regard to incarcerated young offenders in Australia, Ashkar and Kenny (2008) found that the adolescents mainly had negative experiences (e.g., antagonism) regarding the contact with youth workers. Such negative perceptions of treatment might have negative consequences for the outcomes of care (cf. Kromhout, Eldering, & Knorth, 2000; Schubert et al., 2012).

These findings suggest that staff should be more responsive to the perspectives of the adolescents. Research has also shown that good responsiveness by professionals is essential for a positive adolescent-staff relationship, which eventually is associated with successful outcomes of secure residential care (Harder et al., 2012, 2013; Holmqvist, Hill, & Lang, 2007). To improve the responsiveness of professionals in secure residential care, training and support seems crucial. Therefore, we recommend investigating the implementation and outcomes of training programs for professionals in secure residential youth care services.

## Limitations

A first limitation of the present study is that it is based on relatively small subsamples. The adolescent sample included adolescents aged 15–20, while secure residential youth care institutions in the Netherlands serve adolescents between the ages of 12 and 23 years. Moreover, only civilly placed girls and mainly criminally placed boys were included, while girls occasionally also stay by criminal court order and boys commonly by civil court order. Therefore, the results may not generalize to all adolescents in the population.

Secondly, the interview data was assessed and analyzed by the first author only. We did not apply independent coding and consequently, we could not calculate interrater reliability between coders. This approach limits the trustworthiness of the results. On the other hand, the highly structured interviews that were used for all respondents provided a clear global division of topics. In addition, the first author both conducted and analyzed all interviews using the same procedure.

Third, the interviews that we used contained some questions that might have been formulated too broadly, resulting in too diverse information. For example, the care professionals might have had more corresponding answers if the question about treatment aspects causing positive change with the adolescents was formulated more specifically. However, we intentionally formulated most questions broadly since it concerns an exploratory study.

A fourth limitation is that we only used interviews as a basis for information about adolescents’ behavior changes during care. By using the interviews we were able to identify aspects within the care process that are considered important in achieving positive behavior changes. However, a more in-depth analysis is needed to serve as a basis for analyzing how residential care actually contributes to behavioral change. Such in-depth analysis can consist of, for example, observations of interactions between adolescents and professionals during residential care and the application of a dynamic systems approach in studying this topic.

## Implications

Despite these limitations, the findings of the present study highlight the importance of good responsiveness of staff to the perspectives of the adolescents and parents in secure residential care. This suggests that more attention to support and training of professionals in secure residential youth care is desirable and necessary, both in research and practice. In this regard, one relevant option for improving professionals’ responsiveness is to train professionals in Motivational Interviewing (MI) techniques (Doran, Hohman, & Koutsenok, 2011; Hohman, Doran, & Koutsenok, 2009). MI seems relevant given that adolescents and parents in the present study recognize the importance of the adolescent’s own responsibility in achieving a positive change. Overall, the results suggest that training should focus on improving those skills that are perceived as important for a positive adolescent-staff relationship, including good listening skills that are often mentioned as characteristics of good professionals by adolescents in the present study.
